# Sleep Disturbance Phenotype Trajectories and Associated Multimorbidity in Children With New-Onset Seizures Over 36 Months

**DOI:** 10.1016/j.pediatrneurol.2025.05.009

**Published:** 2025-05-12

**Authors:** Temitayo Oyegbile-Chidi, Sabrina Tu, Jordan Eisner, Danielle Harvey, David Dunn, Jana Jones, Anna Byars, Bruce Hermann, Joan Austin

**Affiliations:** a Department of Neurology, University of California Davis, Sacramento, California; b Department of Neurology, University of California San Diego, San Diego California; c Department of Public Health Sciences, University of California Davis, Davis, California; d Departments of Psychiatry and Neurology, Indiana University, Indianapolis, Indiana; e Department of Neurology, University of Wisconsin School of Medicine and Public Health, Madison, Wisconsin; f Department of Pediatrics, Cincinnati Children’s Hospital at the University of Cincinnati, Cincinnati, Ohio; g Distinguished Professor Emerita, School of Nursing, Indiana University, Indianapolis, Indiana

**Keywords:** Epilepsy, Seizures, Pediatric, Sleep Cognition, Behavior, Sociodemographic disadvantage

## Abstract

**Background:**

Children with epilepsy exhibit heterogeneity and variability in comorbidities including sleep disturbance, cognitive dysfunction, and behavioral impairments. This heterogeneity raises the question of whether there are unique sleep phenotypes associated with epilepsy. Here we investigate the presence and progression of sleep phenotypes in youth with new-onset epilepsy over a three-year period and, in addition, characterize their associated cognitive and behavioral profiles to inform the presence and degree of multimorbidity.

**Methods:**

A total of 312 subjects (aged 6–16 years) were recruited within six weeks of their first recognized seizure. Sleep was evaluated in each child using the Sleep Behavior Questionnaire, assessing five areas of sleep disturbance—bedtime difficulties, parent-child interactions, sleep fragmentation, parasomnia, and daytime drowsiness. Each child also underwent a comprehensive neuropsychologic and behavior assessment. All sleep, cognitive, and behavior measures were evaluated at baseline, 18 months later, and 36 months later.

**Results:**

Latent trajectory analysis over the 36-month period identified three sleep phenotypes: a sleep phenotype similar to healthy control subjects (*Typical Sleep* phenotype), a *Moderately Disrupted* sleep phenotype, and a *Severely Disrupted* sleep phenotype. The *Typical* phenotype exhibited the least sleep disturbance overall with the lowest levels of cognitive and behavior problems. The *Moderately* and *Severely Disrupted* sleep phenotypes exhibited more sleep disturbance overall and showed the highest levels of associated cognitive and behavioral dysfunction.

**Conclusions:**

Sleep disturbance phenotypes in youth with new-onset epilepsy are stable and persistent. These distinct phenotypes are also associated with unique cognitive and behavioral patterns—indicating the presence of substantial multimorbidity.

## Introduction

Childhood epilepsy is a multifaceted condition often associated with significant comorbidities, including disturbances in sleep, cognition, behavior, socialization, and academic performance.^1–6^ These complications collectively highlight the broader impact of epilepsy on quality of life and developmental trajectories. Historically, these challenges have been studied as isolated phenomena, typically using cross-sectional designs that compare individuals with epilepsy with control groups. Although these studies provide valuable insights, they often paint a bleak picture of childhood epilepsy and fail to capture the heterogeneity of individual experiences.

Recent advancements emphasize the utility of phenotype-based approaches to better delineate the diverse presentations of childhood epilepsy. Sleep disturbances, for example, exhibit substantial variability, ranging from patterns comparable to healthy control subjects to severe disruption.^2,6,7^ Furthermore, phenotypic clustering allows for the identification of distinct subgroups, revealing heterogeneity in the severity and trajectory of these disturbances.^8–10^ This approach moves beyond the traditional cross-sectional framework, offering a more nuanced understanding of individual differences and their longitudinal progression.

Moreover, the study of epilepsy-related comorbidities has largely been conducted in silos, with separate streams of research focusing exclusively on cognition, behavior, or sleep.^3,11–14^ Although this segmented approach allows for deep exploration of specific issues, it often overlooks the multimorbidity problem, where multiple complications coexist and interact. Although there is accumulating evidence that these comorbidities are inter-related,^1,2,4–7,15,16^ the aggregation of these challenges, particularly the co-occurrence of sleep disturbances with cognitive and behavioral dysfunctions, remains understudied^6^ but is critical for understanding the broader impact of epilepsy on brain development.

Understanding the temporal course of these complications is equally important. Longitudinal studies offer essential insights into whether these issues improve, worsen, or remain stable over time. There is consensus that many complications of epilepsy, including sleep disturbances, are apparent at the time of diagnosis or even before the onset of seizures.^1,2,4–7,15,16^ Studying new-onset epilepsy with a longitudinal approach thus provides a unique window into early intervention opportunities.

Sleep disturbances in particular warrant focused attention, as they are among the most common and persistent complications in children with epilepsy. Beyond their standalone impact, sleep problems often co-occur with cognitive and behavioral challenges, forming a constellation of multimorbidities that may exacerbate the overall burden of epilepsy. Although causality cannot be inferred, understanding these patterns of co-occurrence is critical for comprehensive management. Additionally, identifying predictors of these phenotypes, including both clinical epilepsy factors and social determinants of health (SDOH), could inform tailored intervention strategies.

Here, our study aims to address critical gaps in the literature through three objectives: (1) compare and contrast cross-sectional versus phenotype-based approaches to characterize sleep problems and their longitudinal course in children with new-onset epilepsy, (2) determine whether sleep phenotypes are a unique comorbidity or part of a broader multimorbidity pattern involving cognitive and behavioral challenges, and (3) identify predictors of sleep phenotypes, incorporating clinical epilepsy characteristics, familial factors, and SDOH dimensions.

By addressing these aims, this study seeks to provide a comprehensive understanding of sleep disturbances and their interplay with other complications in children with epilepsy, informing future research and intervention efforts.

## Methods

### Participants

Study participants included children with newly diagnosed seizures and their primary caregivers in each household.^17,18^ The core investigation was conducted at Indiana University and Cincinnati Children’s Hospital at the University of Cincinnati. A total of 312 children were recruited within six weeks of their first recognized seizure (mean = 35 days). Children were recruited through electroencephalography (EEG) laboratories, emergency departments, and pediatric neurologists in two large children’s hospitals (Indianapolis and Cincinnati) and from practices of private pediatric neurologists in Indianapolis. The Cincinnati recruitment site provided the most subjects from their busy first-seizure clinic. The Indiana recruitment site recruited newly-referred children from the general epilepsy clinics. When children met the criteria, refusals were less than 10%. All children in this sample met the International League Against Epilepsy criteria for epilepsy.^19^

Exclusion criteria were a comorbid chronic physical disorder, intellectual disability (based on either clinic records or parent report), or seizures precipitated by an acute event (e.g., intracranial infection, metabolic derangement, and recent head injury). Children who had had two or more febrile but no afebrile seizures or who were placed on daily antiseizure medication (ASM) after a febrile seizure were also excluded. Parental informed consent and child assent were obtained before data collection. The study was approved by the institutional review boards at Indiana University and Cincinnati Children’s Hospital Medical Center.

Data were first collected within six weeks of the first recognized seizure (baseline; B) from the children with newly diagnosed seizures. All participants were followed prospectively and reassessed 18 months later (M18) and finally, 36 months later (M36). The attrition rate over the first 18 months of the investigation was 10% and another 5% over the second 18 months. All data were included in the analysis regardless of the number of visits completed.

### Instruments

#### Sleep evaluation

The Sleep Behavior Questionnaire (SBQ) was completed by the parent to characterize the child’s sleep problems during the prior six months (B), 18 months later (M18), and 36 months later (M36). Details of this instrument are provided elsewhere.^1,5^ Briefly, the SBQ has 35 items describing sleep habits and behaviors that are rated using five-point scales of 1 (*never*), 2 (*just a few times*), 3 (*sometimes*), 4 (*quite often*), 5 (*always*). Parents were specifically instructed to exclude any behaviors that might have been actual seizure activity or any unusual sleep behaviors that occurred immediately before, during, or after a seizure episode. The reliability and validity of the SBQ as well as norms based on behavior and age have been established in the past.^1,2^ This study focused on the summary scores for bedtime difficulties, parent-child interactions, sleep fragmentation, parasomnia, and daytime drowsiness. The final score varies between 26 and 130. The higher the score, the greater the number of sleep problems, which consequently indicates worse sleep disturbance overall. In prior studies, average sleep problems in epilepsy scored at 54 and average sleep problems in healthy control subjects scored at 38.2.^1^

#### Cognitive evaluation

All children completed a comprehensive neuropsychologic evaluation that included standardized clinical measures of intelligence, language, immediate and delayed verbal and visual memory, executive function, speeded fine motor dexterity, and academic achievement at baseline, M18, and M36. The specific administered tests included Clinical Evaluation of Language Fundamentals, 3^rd^ Edition^20^; Comprehensive Test of Phonological Processing^21^; Conners’ Continuous Performance Test, 2^nd^ Edition^22^; Kaufman Brief Intelligence Test^23^; Coding and Symbol Search Subtests of the Wechsler Intelligence Scale for Children, 3^rd^ Edition^24^; Wide Range Assessment of Memory and Learning Design Copy^25^; and the Wisconsin Card Sorting Test.^26,27^ Testing was administered by psychometrists who were trained, observed, and certified on the test battery and its scoring by a pediatric neuropsychologist.^26,28^

Each test was administered according to standardized procedures, and scores were converted to age-corrected standardized scores using the best available national norms for all tests except Wide Range Assessment of Memory and Learning Design Copy, which was designed by this study’s research group; this test was normed internally, using our own sample to generate age-corrected scores. Factor analysis of these neuropsychologic test data revealed four underlying factors: (1) Language, (2) Processing Speed, (3) Executive Function/attention/construction, and (4) Verbal Memory and Learning.^2,29^ The Language factor consisted of measures of verbal concept formation, phonological awareness, and phonological memory. The Processing Speed factor consisted of measures assessing psychomotor speed and rapid naming. The Executive Function factor consisted of measures assessing sustained attention, problem solving, and visual construction. The Verbal Memory and Learning factor consisted of measures of rote verbal learning and story recall. Higher factor scores indicate better neuropsychologic performance.^28^

#### Behavioral evaluation

Two instruments were used to assess emotional and behavioral concerns: (1) Child Behavior Checklist (CBCL) completed by parents and (2) Teacher Report Form (TRF) completed by teachers.^30^ Relevant details follow below.

##### CBCL and TRF.

The CBCL was completed by a caregiver/parent to measure each child’s behavior problems during the past six months, the test administered at baseline (B), M18, and M36. Details of this instrument are provided elsewhere.^30^ Briefly, the CBCL has 118 items describing behaviors that are rated using three-point scales of 0 (*not true*), 1 (*somewhat or sometimes true*), and 2 (*very true or often true*).^30^ Three summary scores from the CBCL were used including T-scores for Total Behavior Problems, Total Internalizing Problems, and Total Externalizing Problems, all normed for age and sex. For the children with seizures, parents were specifically instructed to exclude any behaviors that might have represented actual seizure activity or any behaviors that occurred immediately before, or after, a seizure. The TRF was completed by each child’s teacher based on the child’s behavior within the past two months at baseline (B—to assess baseline emotional-behavioral status and affect) and again at the M18 and M36 time periods (18 and 36 months following the child’s first seizure). Details of this instrument are also provided elsewhere. The TRF was completed by one teacher only (primary teacher) per time period, who usually was a different primary teacher at each time period. Like the CBCL, each item was rated on a three-point scale, and scores were computed for the three broadband scales: Total Behavior Problems, Internalizing Problems, and Externalizing Problems.

Both the CBCL and TRF have been used extensively in children with epilepsy and have been found to be reliable and valid in the pediatric epilepsy population.^13,16,18,31,32^ Many past studies have relied primarily upon parents to rate their child’s behavior problems. Making use of both the CBCL and TRF provides insight into informant consistency and lends credence to the reliability of the behavior problems of the child as seen in multiple different settings (school and home primarily).

Testing was administered by psychometrists who were trained, observed, and certified on the test battery and its scoring by a pediatric neuropsychologist.^26^ All seizure characteristics and sociodemographic data (e.g., caregiver’s highest education level, caregiver’s household income, child’s age, child’s sex, and child’s education) were collected via structured interviews by trained research coordinators as well as psychometrists. Clinical seizure variables including seizure classification and results of EEG and imaging were collected from the electronic medical record and were coded independently by study physicians unaware of the behavioral or cognitive data. The sociodemographic data, collected at the baseline (B) visit only, included highest level of maternal education, household income, parental marital status, and self-identified race.

### Statistical analysis

#### Cross-sectional group comparisons

Statistical Package for Social Sciences (SPSS) software (Version 29.0, IBM, Chicago, IL, USA) was used. One-way analysis of variance tests compared sleep disruption phenotype differences as well as sleep phenotype by cognitive testing and behavioral performance at each time point (B, M18, M36). When the F statistic was significant, Tukey honest significant posthoc comparisons were conducted among the sleep phenotypes.

#### Longitudinal group comparisons: latent trajectory modeling

To identify distinct patterns of sleep disturbance in a large sample of children with new-onset epilepsy over a 36-month period, the summary sleep score was included in an analysis of latent group-based trajectory modeling (LGBM) of longitudinal data, which was carried out by SAS *Proc Traj*.^33^ LGBM captures the heterogeneity of subgroups among a specific population by simultaneously estimating several trajectories as opposed to fitting an overall population mean. For each participant, the summary sleep score was collated for each child at each visit (B, M18, and M36). To find the optimal number of trajectories, Bayesian information criterion compares the fitness of models between trajectories with a differing number of groups or between different shapes of a trajectory. At a baseline, we ensured a minimum of 10% for each of the trajectory groups. *PROC SURVEYLOGISTIC* was utilized to identify the significant risk factors for each of the sleep phenotypes in the univariate analysis. The level of significance *P* < 0.05 was used for the multivariate logistics regression. LGBM data analyses were all conducted using SAS version 9.4.

The risk factors assessed in the analyses included clinical seizure characteristics (epilepsy syndrome [0 = primarily generalized, 1 = localization-related], EEG results [0 = normal, 1 = abnormal], magnetic resonance imaging results [0 = normal, 1 = abnormal], neurological examination at baseline [0 = normal, 1 = abnormal], age of onset of first recognized seizure, seizure frequency [number of seizures/year], and percent on first ASMs) as well as demographic characteristics (age, sex, grade, handedness, and sociodemographic score). The Sociodemographic Disadvantage Score is an index based on four sociodemographic variables—mother’s education level, race (self-identified), household income, and marital status. Details are provided elsewhere.^34,35^ Briefly, families were assigned a rating based on disadvantage level. For caregiver education level and household income, those families below the mean were assigned a score of *0*, whereas those families at or above the mean were assigned a score of *1*. For race and caregiver marital status, nonwhite race and nonmarried status were each assigned a score of *0*, whereas white race and married status received a score of *1*. Scores ranged from 0 to 4 with lower scores indicating higher disadvantage.

## Results

### Sample characteristics

Table 1 summarizes the demographic and clinical seizure characteristics for the sample. Briefly, a total of 312 children with newly diagnosed seizures aged 6–16 years were included in the analyses. The clinical epilepsy characteristics indicate an average age of onset of seizures of 9.42 years, and ~40% of the seizure group was composed of localization-related epilepsy syndromes. At the baseline visit, 12.3% of children with epilepsy had been started on ASMs, as children were recruited after epilepsy diagnosis. The five most frequently prescribed ASMs were lamotrigine, oxcarbazepine, carbamazepine, phenytoin, and valproic acid. Other less commonly prescribed medications included levetiracetam, ethosuximide, zonisamide, and gabapentin. The epilepsy syndromes were divided into two groups: primary generalized (generalized tonic-clonic, absence, and myoclonic epilepsy syndromes) and focal/localization-related (focal unaware and focal aware seizures with or without secondary generalization). In this cohort, magnetic resonance imaging abnormalities included multiple various abnormalities (e.g., bilateral or unilateral hippocampal atrophy/sclerosis, ventricular enlargement, volume loss, cortical dysplasias, heterotopias, angiomas, encephalomalacia, and old hemorrhages) as described in detail elsewhere.^26^ The EEG abnormalities included focal and generalized epileptiform activity (localized and generalized intermittent slowing, continuous slowing, epileptiform discharges, electrographic seizures, occipital intermittent delta activity, and frontal intermittent delta activity). In this cohort, 62% evidenced epileptiform activity, 11% slow wave activity, and 1% electrographic seizures.^28^

### Cross-sectional group comparisons

#### Sleep disturbance over three years

**FIGURE 1.**
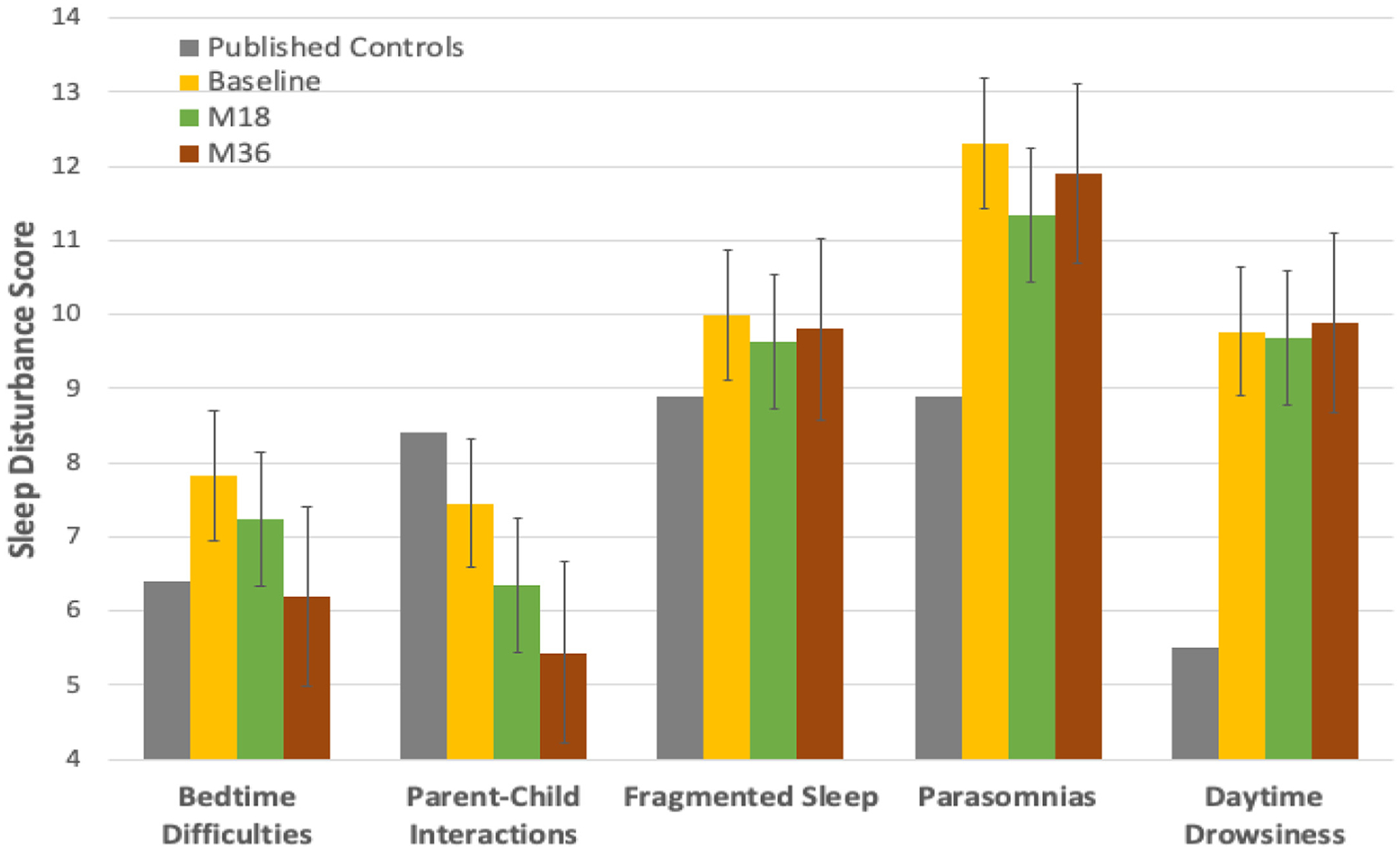
Sleep disturbance over a three-year period. Children with new-onset seizures exhibit higher sleep disturbance compared with reference control subjects (gray bars). Fragmented sleep, parasomnias, and daytime drowsiness are most affected. This pattern remains persistent over the three visits. Behavioral sleep problems—bedtime difficulties and parent-child interactions—reduce over time as expected with child maturation (higher total sleep scores indicate higher levels of sleep disturbance).

A comparison of children with seizures and control subjects showed that sleep disturbance in children with new-onset epilepsy was on average substantially higher compared with published reference control subjects.^1^ Specifically, children with new-onset seizures experienced higher levels of fragmented sleep, parasomnias, and daytime drowsiness consistently over a three-year period compared with reference control subjects (Fig. 1). However, as expected with child development, behavioral sleep problems—bedtime difficulties and parent-child interactions—reduce over a three-year period, reflecting age-related developmental improvements in sleep regulation and behavior.

#### Longitudinal group comparison: sleep phenotype clusters

**FIGURE 2.**
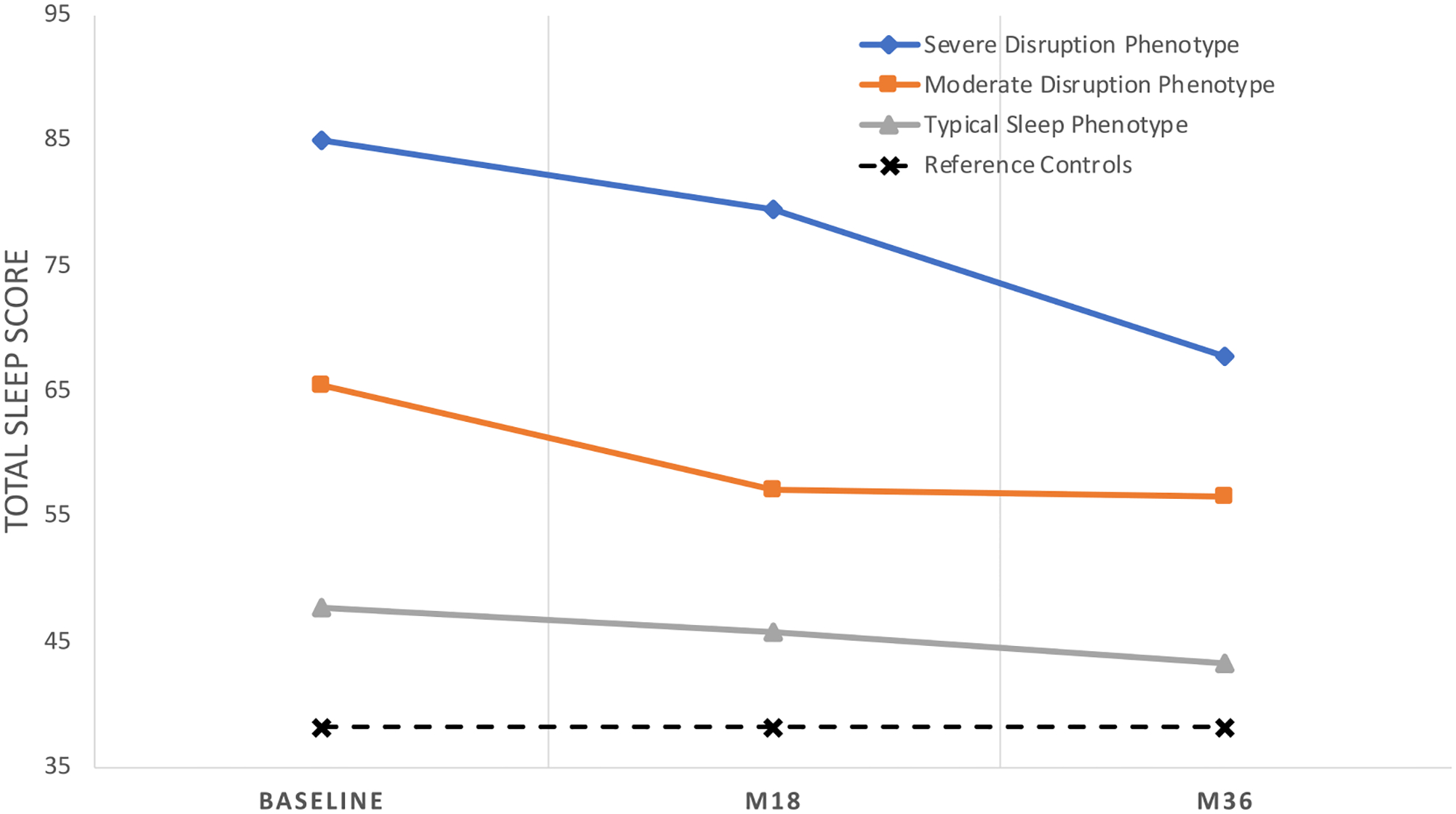
Latent sleep phenotypes trajectories. Latency trajectory analysis from the three time points (baseline, M18 [18 months from baseline], and M36 [36 months from baseline]) resulted in three distinct phenotype clusters. The *Typical Sleep phenotype* exhibited sleep similar to control subjects over the three-year period, whereas the *Moderately Disrupted Sleep* and *Severely Disrupted Sleep* phenotypes exhibited consistently higher levels of sleep disruption compared with control subjects (higher total sleep scores indicate higher levels of sleep disturbance).

Upon further investigation using latent trajectory analysis, three distinct sleep phenotypes were established (see Fig 2). The *Typical phenotype* (69.4%) showed the lowest levels of sleep disturbance, which was closest to that of control subjects. On the other hand, the *Severely Disrupted phenotype* (16.9%) showed the highest levels of sleep disturbance overall, whereas the *Moderately Disrupted phenotype* (13.7%) showed a consistently moderate level of increased sleep disturbance over the 36-month period. There were significant effects of phenotype group and time point (F(1,308) = 240.3, *P* < 0.001, η = 0.539) with significant univariate effects using Tukey significant difference across time points (all Tukey *P* values < 0.001).

#### Sleep phenotypes and associated multimorbidity

Owing to the relatively small sample sizes of the *Moderately* and *Severely Disrupted Sleep phenotypes*, the severely and moderate phenotype groups were combined into a *Disrupted* phenotype for further investigations.

#### Intersection between sleep phenotypes and cognitive outcomes

**FIGURE 3.**
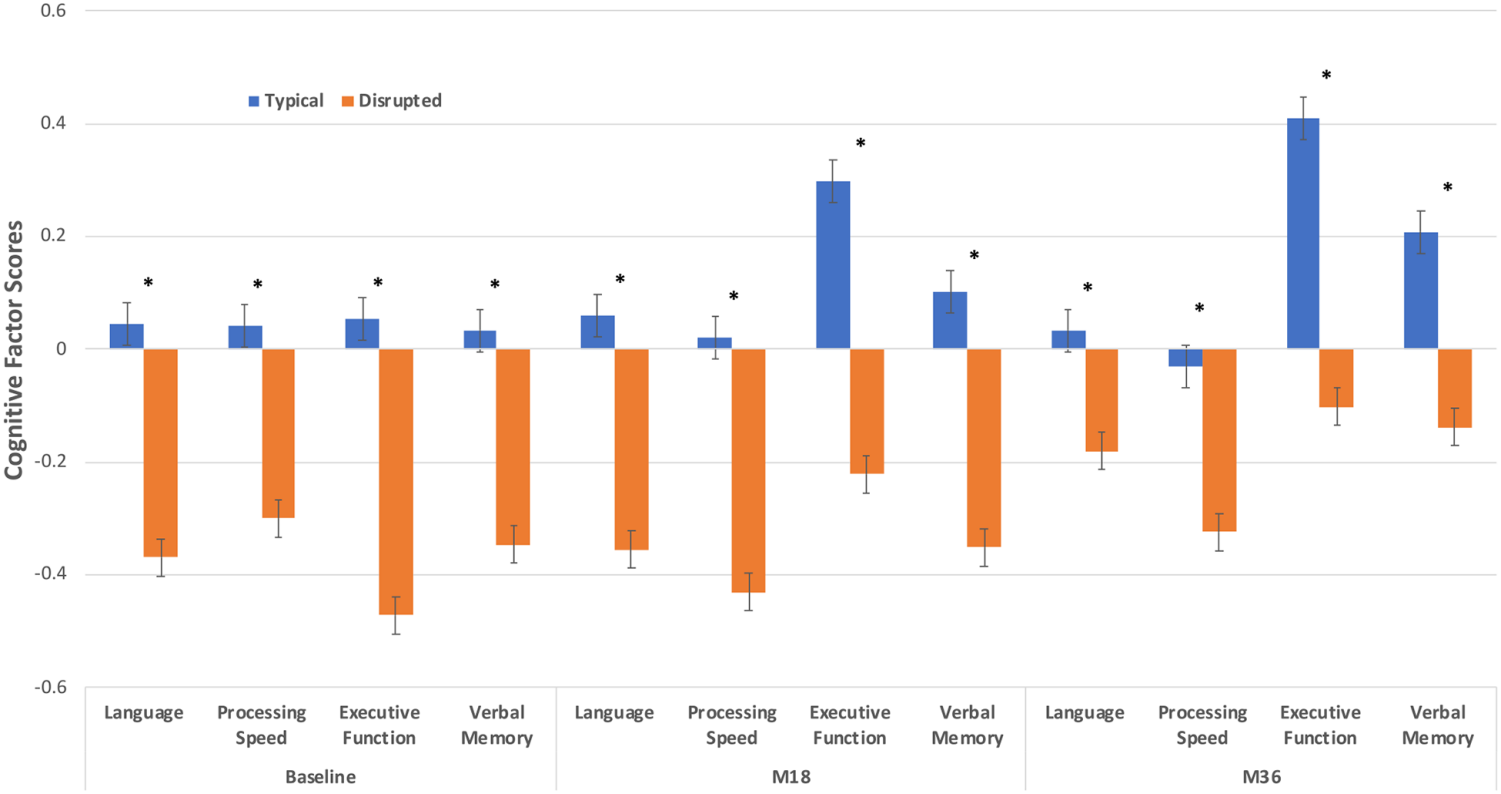
Intersection between sleep phenotypes and cognition over a three-year period. Children in the *Typical Sleep* phenotype exhibit significantly better performance than those in the *Disrupted Sleep* phenotype. This pattern remains persistent and significant over all the visits (*P < 0.05).

There was a notable relationship between sleep and cognition as the distinct sleep phenotypes exhibit unique cognitive signatures as revealed by comprehensive neuropsychologic assessment (F(1,270) = 3.12–9.69, all *P value*s < 0.05) (Fig 3). On average, children in the *Disrupted* sleep phenotype show higher levels of cognitive dysfunction, generally affecting all cognitive domains. On the other hand, the *Typical* sleep phenotype showed lower levels of cognitive dysfunction. These findings were consistently significant over a 36-month period.

#### Intersection between sleep phenotypes and behavioral outcomes

**FIGURE 4.**
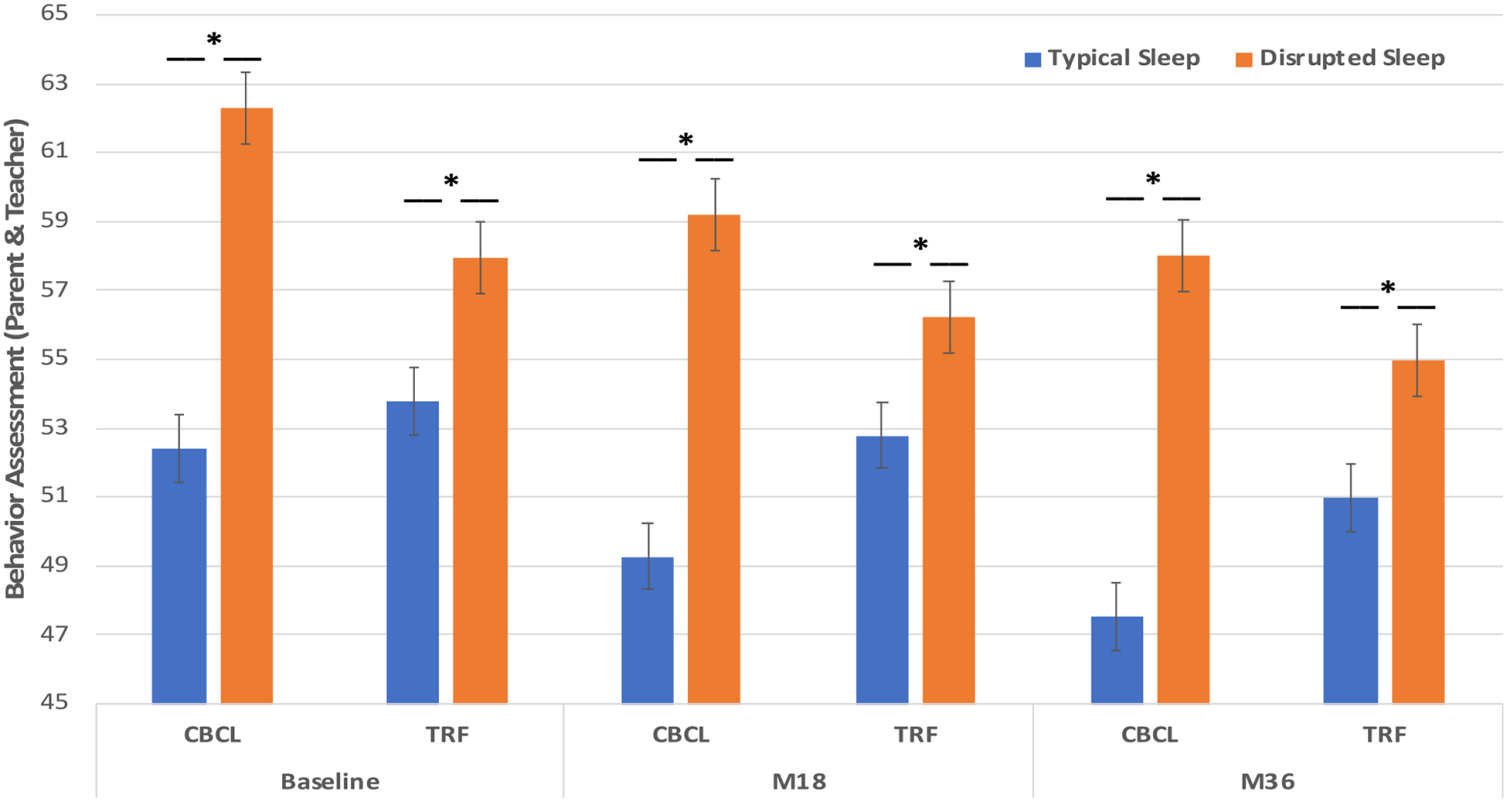
Intersection between sleep phenotypes and behavioral outcomes over a three-year period. Children in the *Typical Sleep* phenotype exhibit significantly lower levels of behavior problems as reported by parents and teachers compared with those in the *Disrupted Sleep* phenotype. This pattern remains persistent and significant over all the visits (all *P* < 0.05). CBCL, Child’s Behavior Checklist, Parent’s Report; TRF, Child’s Behavior Checklist, Teacher Report Form.

There was also a notable relationship between sleep and behavior outcomes as the distinct sleep phenotype groups showed distinct behavioral patterns as reported by both parents and teachers (F(1,308) = 5.69–59.35, all *P*’ values < 0.05) (Fig 4). On average, children with seizures in the *Disrupted Sleep* phenotype exhibited significantly higher levels of behavioral problems compared with those in the *Typical Sleep* phenotype. Again, these findings were consistently significant over a 36-month period.

#### Predictors of phenotype class membership

Using multinomial logistical regression analyses, we determined which baseline risk factors best predict *Typical* or *Disrupted Sleep* phenotype class membership (Table 2). Only sociodemographic disadvantage played a significant role in predicting class membership.

## Discussion

The goal of this study was to extend prior findings indicating heterogeneity of sleep problems in children with new-onset epilepsy.^6^ Prior work in our laboratory has noted the inter-relationship between sleep, cognition, and behavior in children with new-onset epilepsy.^5,36^ We extend those findings here by highlighting the significance and persistence of sleep disturbance in youth with new-onset seizures and its broader implications for cognitive and behavioral outcomes in these children, using advanced analytical techniques. Our findings underscore critical inter-relationships between sleep disruption, cognitive, and behavioral factors along with associated sociodemographic and clinical factors.

In general, children with new-onset seizures consistently exhibit higher levels of sleep disturbance—particularly characterized by fragmented sleep, parasomnias, and daytime drowsiness—compared with published reference control subjects. These findings align with prior literature that implicates seizures in disrupting typical sleep patterns.^1,5,37^ However, when analyzed in more detail, using machine learning three distinct sleep phenotypes emerged, each with unique cognitive and behavior signatures. These phenotypes remained stable over the three-year follow-up, with the disrupted groups consistently demonstrating greater levels of sleep disturbance. Overall, our findings indicate that youth with a disrupted sleep trajectory pattern had a higher likelihood of having more cognitive and behavioral challenges as well as having a more disadvantaged background. On the other hand, those with a more typical sleep trajectory pattern had a higher likelihood of having fewer cognitive and behavioral challenges as well as having a less disadvantaged background.

The cognitive and behavioral assessments provide compelling evidence for the downstream consequences of sleep disruption in children with epilepsy. Furthermore, these findings reinforce the bidirectional relationship between sleep and neuropsychologic outcomes, with chronic sleep disturbances likely exacerbating the cognitive and behavioral challenges already inherent to childhood epilepsy.

Notably, sociodemographic disadvantage emerged as the most significant predictor of disrupted sleep phenotype membership. Children from more disadvantaged backgrounds were disproportionately represented in the *Disrupted* phenotypes, highlighting the critical role of environmental and socioeconomic factors in moderating the clinical trajectory of epilepsy. This finding underscores the importance of addressing SDOH in the management and care of children with epilepsy.

A significant and noteworthy finding is that a relatively high proportion of youth with seizures—over two thirds of the studied sample—exhibited minimal sleep disturbances. This observation provides an encouraging perspective, as it suggests that the impact of this disorder on sleep may not be as universally detrimental as previously believed. Highlighting this finding is essential, as it counters the predominantly negative narrative surrounding the disorder, offering a more nuanced and optimistic view. This finding also underscores the importance of identifying factors that contribute to preserved sleep quality in this population, which could guide interventions for those experiencing sleep disruptions.

The stability of sleep phenotypes and their associated cognitive and behavioral outcomes over three years underscores the need for early and sustained interventions to mitigate the impact of sleep disturbances. Targeted strategies, such as behavioral sleep interventions, cognitive behavioral therapy, and family-focused support programs, may help alleviate sleep-related challenges and improve the overall quality of life for children with seizures and their families.

It is important to note that the effects of ASMs may have played a role in our findings as several ASMs can affect sleep, behavior, and cognition adversely. However, the baseline findings reflect sleep and behavior over the six months before the first seizure and cognition before or concomitant with starting ASMs. This fact indicates that there is evidence of sleep, behavior, and cognitive problems independent of ASMs, which is consistent with prior literature that cognition and behavioral challenges can be observed before the diagnosis of epilepsy.^38,39^ In addition, our data collection did not include information on nighttime seizures versus daytime seizures. Future studies evaluating whether increased nighttime seizures are associated with more complaints of parasomnias and fragmented sleep would be critical. The inferences from our study are also limited as our control subjects were based on published reference control subjects. Furthermore, some of the findings were based on subjective data (using well-validated surveys). More advanced evaluations with polysomnogram and computational EEG analysis would be warranted in future studies to further understand the inter-relationships of these multimorbidities with more objective measures.

In conclusion, this study elucidates the pervasive and multifaceted nature of sleep disturbances in childhood epilepsy and their long-term implications for cognitive and behavioral outcomes. Future research should focus on the mechanisms underlying these relationships and explore tailored interventions to optimize sleep health in this vulnerable population.

## Figures and Tables

**TABLE 1.** Sample Characteristics for Children With Seizures

Characteristics	Children With Seizures
**Group characteristics**	
Sample size	312
Child’s age, years (S.D.)	9.44 (2.6)
Child’s sex M/F	158/154
Child’s IQ (S.D.)	100.96 (15.3)
Grade, years (S.D.)	3.79 (2.45)
**Clinical epilepsy characteristics**	
Age of onset, years (S.D.)	9.42 (2.54)
Seizure frequency, per year (S.D.)	43.32 (174.71)
% With FUS (most common seizure type)	41.7% FUS
% With ≥2 seizure types	8.5%
MRI at baseline (% normal)	68.4%
EEG at baseline (% normal)	26.8%
Neurological examination at baseline (% normal)	94.8%
ASMs at baseline (% on ASMs)	12.3%
Handedness (% left handed)	11%
**Household/mother sociodemographic characteristics**	
Self-identified race (% white/Caucasian)	78.8% white
Mean household income (S.D.)	$60,000–70,000 ($27,500)
Mean caregiver education, years (S.D.)	13.82 (2.25)
% Married	76% married
Sociodemographic Disadvantage Score (% disadvantaged)	29.8%

Abbreviations:

ASM = Antiseizure medication

EEG = Electroencephalography

F = Female

FUS = Focal unaware seizures

M = Male

MRI = Magnetic resonance imaging

Data presented as mean (S.D.).

All data at baseline represent the child’s information at the time of the baseline visit, which occurred after epilepsy diagnosis and subsequent study recruitment (income adjusted for today’s dollar value).

**TABLE 2.** Predictors of Sleep Phenotype Class Membership Using Baseline Clinical, Epilepsy, and Sociodemographic Characteristics

Sleep Phenotype Predictors	Standardized β Coefficient	S.E.	*P*	95% CI Lower	95% CI Upper
Typical sleep vs disrupted sleep					
Age	0.442	0.364	0.224	0.763	3.175
Sex (F vs M)	−0.232	0.306	0.448	0.435	1.445
Grade	−0.091	0.244	0.709	0.566	1.473
Age of onset	−0.661	0.440	0.133	0.218	1.223
Seizure burden	−0.001	0.001	0.491	0.998	1.001
Seizure type (Gen vs Foc)	−0.274	0.337	0.417	0.393	1.473
MRI at baseline (normal vs abnormal)	−0.102	0.326	0.754	0.477	1.709
EEG at baseline (normal vs abnormal)	−0.593	0.389	0.127	0.258	1.184
Neurological examination at baseline (normal vs abnormal)	−0.426	0.585	0.466	0.208	2.054
Antiseizure medications at baseline (yes vs no)	−0.212	1.028	0.837	0.108	6.063
Sociodemographic Disadvantage Score	−**1.691**	**0.457**	**<0.001**	**0.075**	**0.451**

Abbreviations:

CI = Confidence interval

EEG = Electroencephalography

F = Female

Foc = Focal seizures

Gen = Generalized seizures

M = Male

MRI = Magnetic resonance imaging

SE = Standard error

Data presented as standardized β coefficients, standard error, and significance, along with confidence intervals. Significance noted in bold.
